# E-scooter related injuries: Using natural language processing to rapidly search 36 million medical notes

**DOI:** 10.1371/journal.pone.0266097

**Published:** 2022-04-06

**Authors:** Kimon L. H. Ioannides, Pin-Chieh Wang, Kamran Kowsari, Vu Vu, Noah Kojima, Dayna Clayton, Charles Liu, Tarak K. Trivedi, David L. Schriger, Joann G. Elmore

**Affiliations:** 1 Department of Emergency Medicine, University of California, San Francisco–Fresno Medical Education Program, Fresno, CA, United States of America; 2 National Clinician Scholars Program, University of California, Los Angeles, CA, United States of America; 3 Department of Emergency Medicine, University of California, Los Angeles, CA, United States of America; 4 Department of Medicine, David Geffen School of Medicine, University of California, Los Angeles, CA, United States of America; 5 Office of Health Informatics and Analytics, UCLA Health, University of California, Los Angeles, CA, United States of America; 6 Veterans Affairs Greater Los Angeles Healthcare System, Los Angeles, CA, United States of America; Indian Institute of Technology Patna, INDIA

## Abstract

**Background:**

Shareable e-scooters have become popular, but injuries to riders and bystanders have not been well characterized. The goal of this study was to describe e-scooter injuries and estimate the rate of injury per e-scooter trip.

**Methods and findings:**

Retrospective review of patients presenting to 180 clinics and 2 hospitals in greater Los Angeles between January 1, 2014 and May 14, 2020. Injuries were identified using a natural language processing (NLP) algorithm not previously used to identify injuries, tallied, and described along with required healthcare resources. We combine these tallies with municipal data on scooter use to report a monthly utilization-corrected rate of e-scooter injuries. We searched 36 million clinical notes. Our NLP algorithm correctly classified 92% of notes in the testing set compared with the gold standard of investigator review. In total, we identified 1,354 people injured by e-scooters; 30% were seen in more than one clinical setting (e.g., emergency department and a follow-up outpatient visit), 29% required advanced imaging, 6% required inpatient admission, and 2 died. We estimate 115 injuries per million e-scooter trips were treated in our health system.

**Conclusions:**

Our observed e-scooter injury rate is likely an underestimate, but is similar to that previously reported for motorcycles. However, the comparative severity of injuries is unknown. Our methodology may prove useful to study other clinical conditions not identifiable by existing diagnostic systems.

## Introduction

In dozens of cities around the world, riders can use a smartphone app to rent an electrically powered stand-up scooter (known as an “e-scooter”) and leave it on the sidewalk at their destination for use by another rider. While some individuals own e-scooters for personal use, most riders rent on a per-mile basis from private operators. The convenience and ubiquity of these personal transporters has led to rapid growth and expansion. As a result, it is estimated that shareable e-scooters may ultimately capture 8–15% of all trips shorter than 5 miles worldwide [1], and reluctance to use other forms of public transportation stemming from the COVID-19 pandemic may further accelerate this trend.

Injuries related to e-scooters were initially largely documented in media reports and led to regulatory reform in some jurisdictions based on limited scientific data on the frequency and types of associated injuries [2]. A previous case review documented 249 patients with injuries resulting in an emergency department (ED) visit connected to e-scooter use during the first year of use in our region (Southern California, more specifically western Los Angeles) [3]. Since then, e-scooter use has increased, and mainstream media has reported on a mounting number of injuries and even fatalities [4, 5], which have prompted individual and class action lawsuits against e-scooter operators and municipalities [6, 7]. As e-scooter operators have expanded, other regions have begun reporting on local injuries, but these reports have been limited to emergency care during initial rollout periods (see **Table 2** in results). No study to date has characterized medical care provided for injuries in a full healthcare system, including in the outpatient setting. Research in this area is hampered by lack of specific diagnosis codes allowing investigators to quickly identify e-scooter injuries. Consequently, the scientific evidence on how much risk e-scooters pose to riders and non-riders remains inadequate.

This study provides the first description of outpatient clinic visits related to e-scooters, in addition to ED and hospital encounters, within a large metropolitan area with widespread e-scooter use. We cover a period five-fold longer than any prior work (**Table 2**), allowing for an assessment of trends over time (including during the ongoing COVID-19 pandemic). This is also the first report, to our knowledge, of downstream related healthcare utilization after patients initially seek care. Given the size of our health system and the larger corpus of detailed clinical data available compared with prior studies, as well as the absence of existing diagnosis codes for injuries resulting from e-scooter use, we applied and tested a natural language processing (NLP) technique to review electronic medical records across millions of visits, a technique not previously used in this area. Furthermore, by merging our injury findings with data reported by e-scooter companies to local municipalities, we also provide an estimate of injury rates on a per trip basis.

## Methods

### Study design overview, setting, and participants

De-identified data were obtained from all patient encounters in the outpatient clinics and two hospitals affiliated with University of California Los Angeles (UCLA) Health. UCLA Health includes an extensive primary care network in the Southern California region providing more than 10 million clinical encounters each year at over 180 locations. Our study population included all patients 10 to 90 years old (in order to exclude patients unlikely to use e-scooters) who had an encounter at an outpatient clinic, ED, or hospital between January 1, 2014, and May 14, 2020. Encounters occurred in a variety of settings, including primary care, all medical specialties, physical therapy, six urgent care centers, and two hospitals with affiliated EDs (UCLA Ronald Reagan and UCLA Santa Monica). Potential cases were identified from the electronic medical record system using an NLP search of all clinical notes and then confirmed by investigator medical record abstraction.

### Definitions

An e-scooter injury was defined as any type of injury attributed in medical records to an electrically powered scooter that led the patient to seek medical care (**S1 Table in S1 File**). This includes a spectrum encompassing major potentially life-threatening traumatic injuries transported by emergency medical services and minor musculoskeletal or skin and soft tissue injuries. Patients involved in e-scooter injuries were further classified as either a rider (e.g., falling off a scooter or hitting an obstacle while riding) or a non-rider (e.g., being hit by an e-scooter or tripping over a parked e-scooter). Cases were further classified based on contextual information in the clinical note as either confirmed e-scooter injury (e.g., when an e-scooter brand name was explicitly stated within the medical records) or possible e-scooter injury (e.g., a 13-year-old child who “fell off a scooter” and is young enough to use a kick scooter, but also old enough to illicitly use a shareable e-scooter).

### Natural language processing (NLP) data labeling and model development

Our NLP technique refines the basic regular expression techniques we developed previously to identify ED visits for e-scooter injuries [3]; we improved this technique over three cycles of training and testing using larger samples of early ED notes prior to 2020. We then tested this algorithm on two holdout sets of notes: ED notes from 2020 and all outpatient notes, neither of which were used for NLP development.

ED visit notes from January 1, 2014 to December 31, 2019 (N = 9,284,161 notes) were used for training the NLP model (**S1 Fig in S1 File**). Cases in our prior study had been identified through a search of ED electronic medical record notes from September 1, 2017 to August 31, 2018 using a regular expression to search for e-scooter keywords, namely the word “scooter” (including “e-scooter”) and two proprietary eponyms (namely, “Bird” and “Lime”, the two most prominent e-scooter brand names at the time of the study). The resulting records, which had all been previously examined in our prior study, were again reviewed and abstracted and yielded 112 confirmed non-cases of e-scooter injury (e.g., records in these non-cases described non-motorized kick scooters or allergies to lime fruit and were clearly unrelated to e-scooters), and 249 confirmed cases of e-scooter injuries (the same number reported in our prior study). These formed the core of our initial training data used for NLP model development.

The initial corpus of training data was then expanded iteratively over three cycles of training and manual review using ED visit records before and after this prior study (January 1, 2014 to August 31, 2017, N = 5,765,950 notes; and September 1, 2018 to December 31, 2019, N = 1,986,120 notes). NLP model 1 added notes matching the term “scooter” in any form, including “e-scooter.” NLP model 2 added notes that included brand names of e-scooter operators (“Bird,” “Lime,” “Lyft,” “Bolt,” “Sherpa,” “Clevr,” and “Hopr”) and common misspellings (“Byrd” and “Lyme”). Finally, to optimize and balance the training data set with negatives, NLP model 3 (the final version) was supplemented with random ED clinician notes for admitted patients that lacked any keywords. All notes used for NLP training and testing data, including negatives, were manually reviewed and verified. A total of 2649 labeled clinical notes, consisting of 1036 confirmed positive e-scooter injuries and 1613 confirmed negatives, as confirmed by manual review, comprised the training and testing data used to generate our final NLP model.

**Fig 1:**
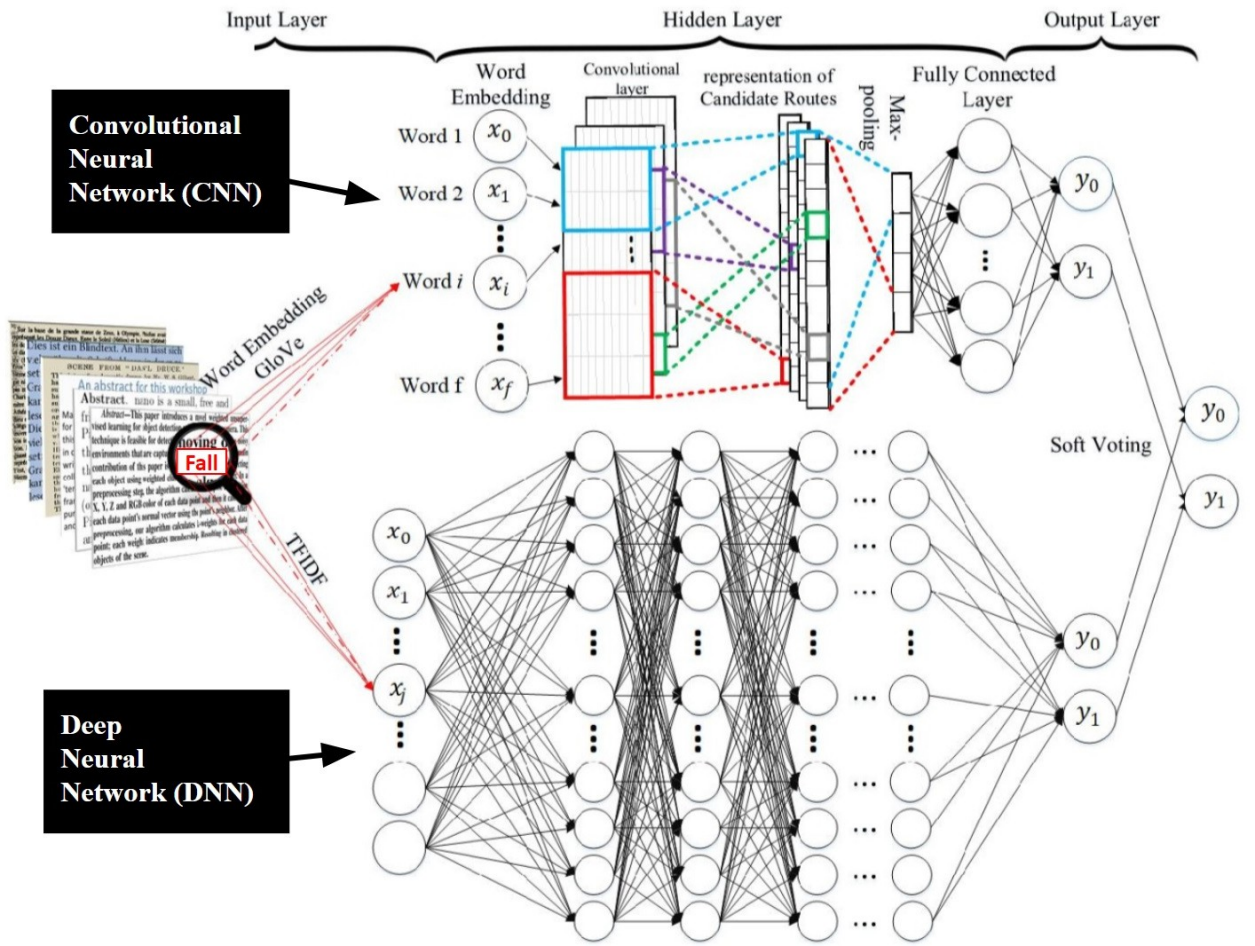
Overview of neural network process for NLP algorithm*. Our process incorporated both convolutional and deep neural networks into an ensemble voting process, known as random multimodel deep learning (RMDL) [8, 9]. This combination of deep learning techniques uses global vectors for word representation (GloVe) [11] input into a convolutional neural network (CNN) and term frequency-inverse document frequency (TF-IDF) input into a deep neural network (DNN), converting language into a series of vectors that convey word meaning and interact to represent combinations of words. Each of two deep learning neural network techniques (CNN at top, and DNN at bottom) then identify patterns in vector representations that correspond to notes in the training data that describe e-scooter injuries, and translate the strength of these patterns into scores from each of the two neural networks that are averaged through soft voting into a probability of e-scooter injury.

At each stage, training data was randomly divided into 70% training and 30% testing subsets. All text in these notes underwent standard NLP pre-processing techniques (tokenization, stop-word removal, and stemming) and were then used to train a binary classifier to identify scooter-related injury in clinical records. We used an ensemble random multi-model deep learning (RMDL) technique (**Fig 1**) [8]. NLP modeling and analysis were performed using Python Version 3.7 with the Keras and TensorFlow packages [9, 10]. The final model achieved an area under the receiver operating characteristic curve of 95% on the full training data (both the 70% training and 30% testing subsets).

### Identification of e-scooter related injuries in testing set

**Fig 2:**
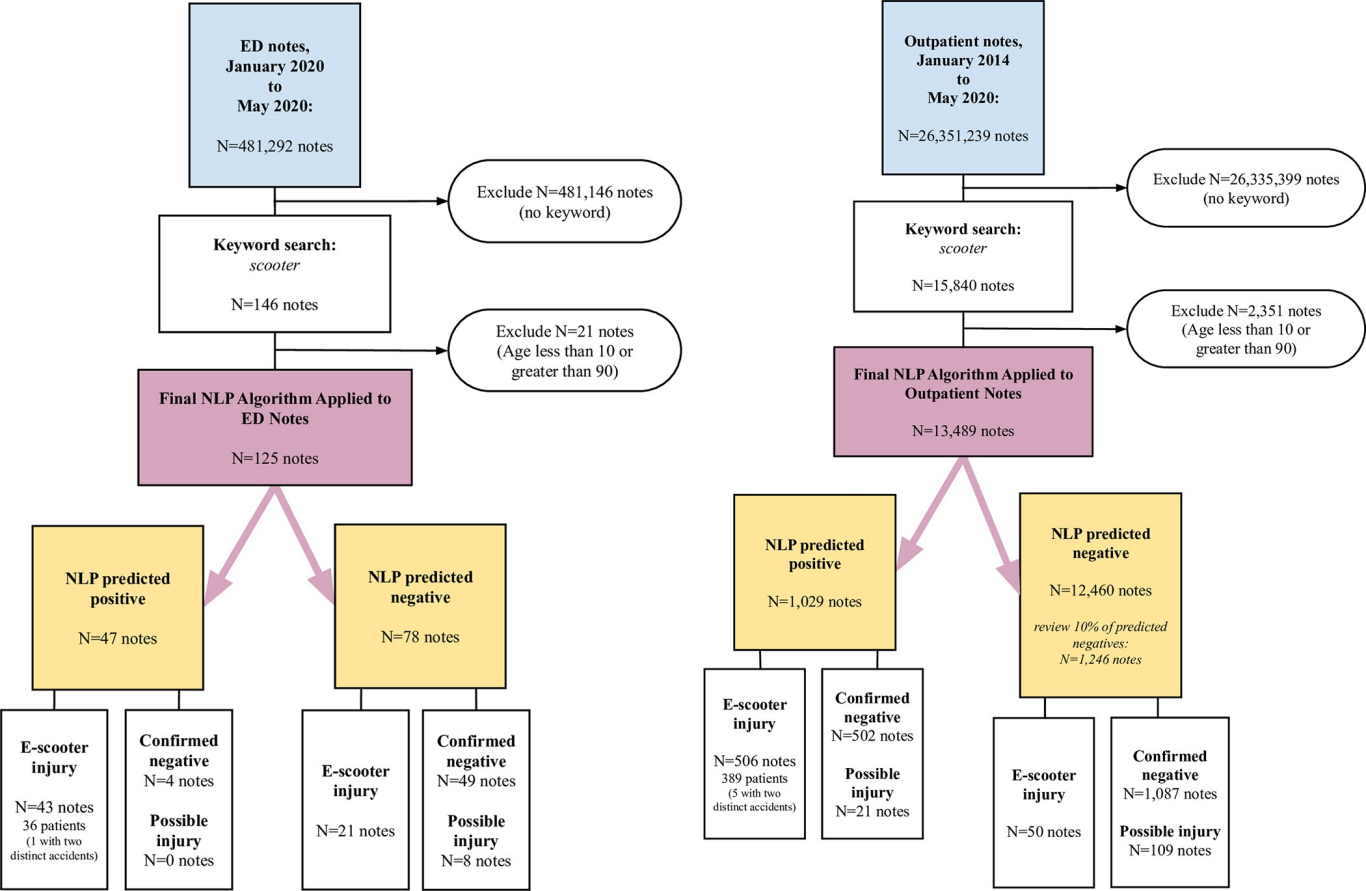
Flowchart of testing process for the predictions of our NLP algorithm*. ED notes in panel A, and outpatient notes in panel B. As in **S1 Fig in S1 File**, “Confirmed Negative” refers to a note that, on review by investigators, did not suffer an e-scooter injury, while “Predicted Negative” refers to a note that our NLP algorithm did not predict as an e-scooter injury. Any cases of possible but not necessarily probable e-scooter injury are not included in our injury tallies, and are treated as non-injuries (negatives) in metrics of diagnostic performance in **S2 Table in S1 File** (as a secondary analysis, we computed and present diagnostic performance under the alternate assumption that possible cases were e-scooter injuries in **S4 Table in S1 File**). Counts refer to numbers of notes, rather than injuries or patients.

The final NLP model was applied to the testing set (**Fig 2**), which included all outpatient visits from January 1, 2014 to May 14, 2020, and ED visits from January 1, 2020 to May 14, 2020 (since ED notes prior to 2020 were already incorporated into our training and testing data). After exclusion of notes from patients under 10 years of age or over 90, the final NLP model was applied to 125 ED notes and 13,489 outpatient notes that contained the keyword “scooter”. An NLP predicted positive was defined as a note with an NLP-predicted probability of e-scooter injury of 90% or greater, while all other notes were defined as NLP-predicted negatives (this threshold was initially set at 50% but later increased in order to improve specificity at the cost of sensitivity and reduce the burden of manual human chart review required–we found that a higher threshold created a set of predicted positives more “enriched” with e-scooter injuries, at the cost of e-scooter injuries “hidden” in a larger set of predicted negatives, which in turn maximized the yield of manual reviews while still allowing for estimation of the false negative rate through a random sample of predicted negatives). All predicted positives were manually reviewed and abstracted by one of the investigators (KLHI, NK, or DC, with any indeterminate cases adjudicated by consensus), allowing for exclusion of notes that did not represent e-scooter injuries and tallying false positives from our model (visits not clearly attributable to an e-scooter injury were classed as non-injuries when there was disagreement or uncertainty among investigators). While all NLP-predicted negative notes from the ED set were reviewed, a random 10% sample of NLP-predicted negative notes were reviewed from the outpatient set (institutional policy limited the total number of notes available for investigator review but allowed our NLP algorithm to scan for scooter injuries without copying data to research databases). This allowed identification of false negatives and estimation of test characteristics against the gold standard of manual review, but false negatives were not included in injury totals.

### Abstraction of index injury and cumulative injury-related medical care

Notes were reviewed to confirm index e-scooter injuries along with 30 days of subsequent medical records in order to abstract cumulative injury-related use of medical resources. Abstraction gathered data on areas of the body injured (head and neck, chest and abdomen, upper extremity, or lower extremity); resources required to evaluate and treat an injury, including clinical visits (inpatient admission, ED, urgent care, outpatient primary or specialty care); procedures (minor, defined as splinting, wound care, or laceration closure, or major, defined as any other procedure that might require sedation or anesthesia); imaging (x-ray, or advanced imaging, including computed tomography, magnetic resonance imaging, or ultrasound); and physical or occupational therapy care. Care for each distinct patient was analyzed as a unit, but evidence of multiple instances of injury in the same patient (i.e., the same patient experiencing multiple e-scooter accidents at distinct times) was noted. Correspondingly, our final injury totals are reported at the patient level, while preliminary reviews to build NLP training data and analysis of NLP diagnostic performance took place at the note level. Finally, year and month of injury was identified based on the recorded index encounter date.

### Calculation of injury rate

Information on shareable e-scooter use in the community was obtained from the city governments of Santa Monica [3] and Los Angeles [12, 13] who, as a condition of permitting shareable e-scooter company operations, mandate reporting of each e-scooter movement, data which is routinely tracked by global positioning system-based sensors inside each scooter. Both cities provided monthly tallies of shareable e-scooter trips in our area in western Los Angeles (as defined by census block groups within our trauma system catchment area, which coincides with the location of most outpatient clinics). Combining these data with our monthly injury counts, we calculate injury rates per trip (e.g., a utilization-corrected injury rate per million e-scooter trips in our catchment area).

### Analysis

Summary statistics and two-sided hypothesis testing employed chi-squared statistics for categorical data, ANOVA for normally distributed data, and Kruskal-Wallis for nonparametric data, with p-values less than 0.05 considered statistically significant, and all confidence intervals computed at the 95% level. SAS 9.4 and Stata 13.1 software were used for all statistical analyses. The UCLA institutional review board approved this study with waiver of informed patient consent (IRB#18-001294-AM-00004). In accordance with the institutional research policies, a small number of patients who requested not to be included in any research were excluded. The study was conducted using the Strengthening the Reporting of Observational Studies in Epidemiology (STROBE) reporting guidelines [14].

## Results

### NLP performance

We searched more than 36 million individual clinical notes during the study period to identify e-scooter related injuries (**S1 Fig in S1 File**). In total, approximately 4 hours of computational time on a standard laptop computer were required, in addition to approximately 1 hour of “wall clock time” for the medical records database to perform a full keyword search. The ED notes from January 1, 2014 to December 31, 2019 were used for a three-stage training and testing process for our NLP algorithm, yielding 1,036 confirmed e-scooter injury notes and 1,613 confirmed negatives after chart review by the investigators (**S1 Fig, panel B in S1 File**).

The final NLP algorithm was then tested on remaining ED notes (from January 1, 2020 to May 14, 2020, N = 481,292 notes) and the outpatient notes from the entire study period (January 1, 2014 to May 14, 2020, N = 26,351,239 notes). After excluding patients aged less than 10 or greater than 90, and excluding notes without the keyword “scooter”, we applied the final NLP algorithm to the remaining 90,824 notes to identify those with a 90% or greater predicted probability of an e-scooter injury. Upon review of all predicted positive notes by investigators, 506 of 1,029 outpatient notes (49%) and 43 of 47 ED notes (91%) were confirmed e-scooter injuries (see **Fig 2**).

E-scooter injuries were noted in 21 of 78 (27%) and 50 of 1246 (4%) of the NLP predicted negative ED and outpatient notes, respectively. The final NLP algorithm had an accuracy of 92%, meaning that our NLP algorithm correctly classified 92% of notes in the testing set with the keyword “scooter” as predictive of e-scooter injury or not. Our algorithm showed greater specificity than sensitivity, which was more pronounced for outpatient notes, which had a lower prevalence of e-scooter injury than ED notes. Overall, a positive likelihood ratio of 12.2 was noted (detailed test characteristics in **S2 Table in S1 File** and confusion matrix in **S3 Table in S1 File**).

The most common false positives noted during training of the NLP model were injuries among pediatric patients using non-electric “push scooters,” typically at home or otherwise away from public roads, and elderly or disabled patients using various assistive devices sometimes referred to as scooters, including “knee scooters” as well as electric wheelchairs. We excluded patients less than 10 or greater than 90 years old in the testing set to reduce the number of such false positives, but they were still noted even after this exclusion.

### Injury information

**Table 1** characterizes all patients with confirmed e-scooter injuries, including both 937 distinct patients from our training data, and 417 additional distinct patients identified by our NLP algorithm during testing. In total, we therefore identify 1,354 distinct patients with e-scooter injuries, most of whom were among e-scooter riders. Our algorithm, however, likely missed roughly 500 injuries that we were unable to characterize that are not included in our tallies (see **S3 Table in S1 File**). Of note, while the NLP identified clinical notes predictive of e-scooter injury, data in **Table 1** are presented at the patient level.

**Table 1:** Characteristics of patients with confirmed e-scooter injuries*.

*Number (percent of total)*	Riders	Non-riders	P-value	Total (N = 1354)
	(N = 1258)	(N = 96)		
**Patient Demographics**				
**Age, years**			< .01	
Less than 18	91(7)	3(3)		94(7)
18–25	398(32)	15(16)		413(31)
26–40	494(39)	24(25)		518(38)
41–64	251(20)	33(34)		284(21)
65 or older	24(2)	21(22)		45(3)
**Sex**			0.17	
Female	539(43)	48(50)		587(43)
Male	719(57)	48(50)		767(57)
**Injury Characteristics**				
**Area of the body**				
Head or neck	533(42)	45(47)	0.39	578(43)
Chest or abdomen	129(10)	13(14)	0.31	142(10)
Upper extremity	691(55)	40(42)	0.01	731(54)
Lower extremity	596(47)	37(39)	0.09	633(47)
More than one area involved	500(40)	33(34)	0.30	533(39)
**Resources Used**				
**Clinical visits**				
Outpatient visit	601(48)	53(55)	0.16	654(48)
Urgent care visit	79(6)	3(3)	0.21	82(6)
Emergency department visit	945(75)	72(75)	0.98	1017(75)
Inpatient admission	72(6)	4(4)	0.52	76(6)
Critical care unit admission	21(2)	1(1)	0.64	22(2)
Visits in multiple settings	376(30)	32(33)	0.48	408(30)
**Imaging**				
X-ray	914(73)	71(74)	0.78	985(73)
Advanced imaging (CT, MRI, US)	361(29)	35(36)	0.11	396(29)
**Procedures**				
Minor (splinting or wound care)	705(56)	41(43)	0.01	746(55)
Major (sedation or anesthesia required)	203(16)	9(9)	0.08	212(16)
**Other**				
Physical or occupational therapy	64(5)	4(4)	0.69	68(5)
Multiple distinct accidents (same patient)	13(1)	1(1)	0.99	14(1)
Substantial resource use	408(32)	34(35)	0.55	442(33)
(visits in multiple settings, or any inpatient admission, major procedure, or physical therapy)				
Death (from trauma, during index visit)	2(0)	0(0)	0.70	2(0)

*All data confirmed by manual clinician review and includes injuries identified in training and testing data. Counts include 937 patients first identified in our training data, which in turn incorporates injuries first identified in our prior study (249 ED visits). P-value displayed is for comparison between e-scooter riders and non-riders (e.g., pedestrians who were hit by a moving e-scooter or tripped over a parked e-scooter). Injuries originally identified from ED notes were recorded as involving a subsequent outpatient visit if any relevant outpatient care was documented in our 30-day review of subsequent visits, and vice-versa for injuries originally identified from outpatient notes. Counts are presented at the patient level, with resource use tallied over the course of all available clinical notes for each patient. If a single patient suffered multiple e-scooter accidents at different times, this was noted, with total resource use totaled across all accidents and injuries (of note, no patients suffered injuries as both a rider and non-rider).

Injured riders were notably younger (P < .01), had more upper extremity involvement (P = 0.01), and received more minor procedures such as splinting or wound care (P = 0.01) compared to injured non-riders. Overall, 39% of patients received care for injuries sustained in more than one body area, 30% were seen in more than one clinical setting for the injury (e.g. initial ED visit followed by an outpatient visit). General X-ray imaging was obtained in 73%, with advanced imaging (CT, MRI, or ultrasound) in 29% and major procedures (requiring sedation or anesthesia) performed in 16% of patients. An inpatient admission was noted for 76 patients (6%), of whom 22 required a critical care unit admission. Two e-scooter riders died as a result of trauma after being hit by motor vehicles. Overall, 33% of patients required substantial resource use for their injuries (defined as a patient with clinical visits in multiple settings, or any inpatient admission, major procedure, or physical therapy use).

### Trends in e-scooter use and injuries

**Fig 3:**
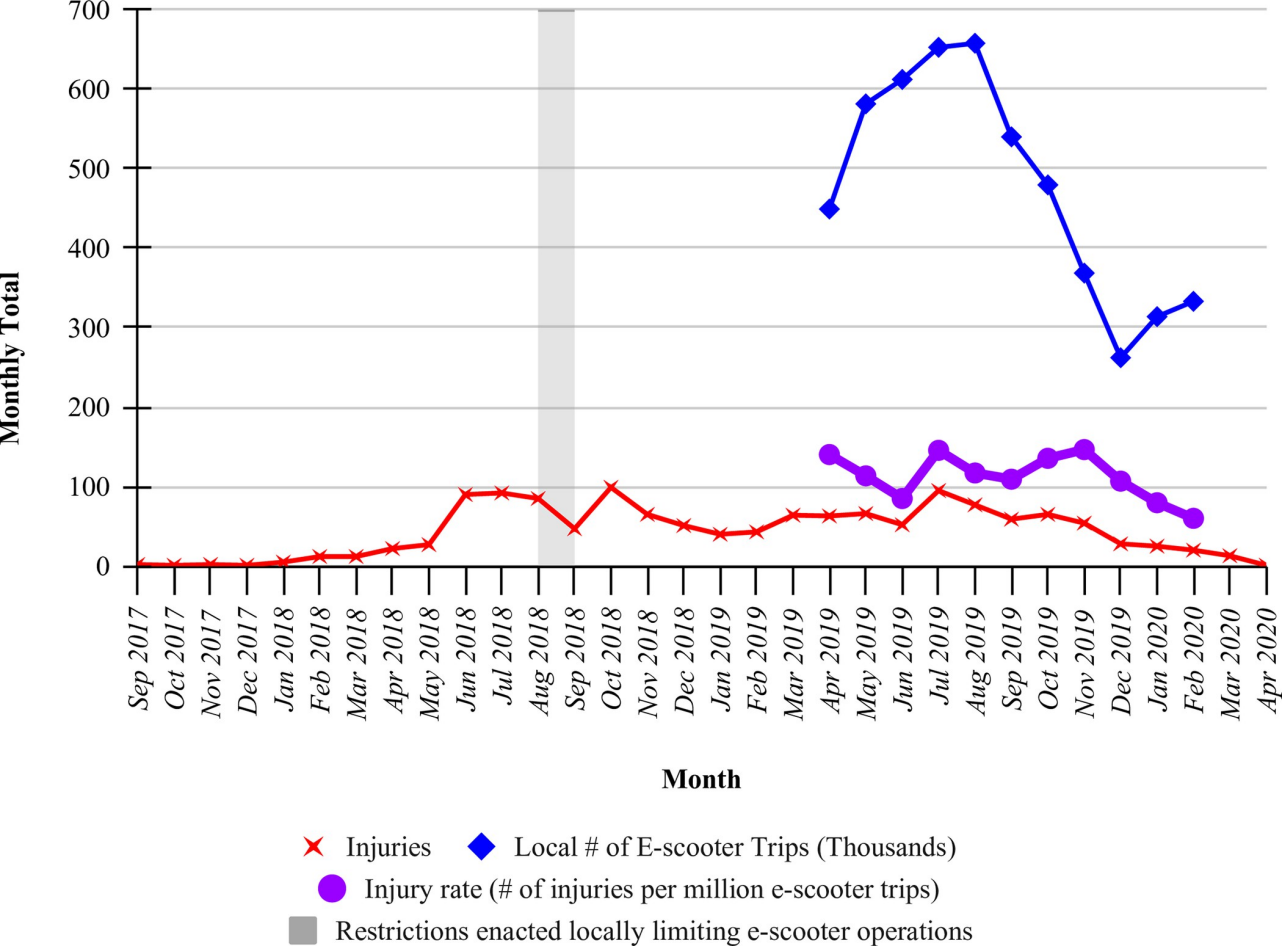
Temporal trends in e-scooter use and e-scooter injuries*. *Availability of e-scooter trip count data for nearby jurisdictions is available for April 2019 until February 2020, and monthly injury rate is therefore only calculable over this period. Note the fall in the number of injuries after a temporary interruption in scooter availability around August 2018 and another fall in injuries during the COVID-19 lockdown in 2020. Months prior to September 2017, which had very few injuries, are not shown here.

Prior to the widespread introduction of shareable e-scooters in 2018, there were at most 13 e-scooter injuries per year (**S5 Table in S1 File**). After introduction of shareable e-scooter operators in our region, e-scooter injuries increased to 595 and 672 in 2018 and 2019, respectively. E-scooter use showed marked seasonal variation (**Fig 3**). During the study period when e-scooter usage data were available for our full catchment area, e-scooter injuries occurred at a mean rate of 54.9 per month, over a mean of 477,209 recorded trips per month, resulting in an overall injury rate of 115 injuries per million e-scooter trips (1 death occurred during this period, corresponding to a rate of 19 fatalities per 100 million e-scooter trips).

## Discussion

In the first study of e-scooter injuries across all outpatient and inpatient care, we identify a growing and concerning absolute number of injuries related to use of e-scooters in our region. Our findings from a dense hub of early e-scooter use may presage long-run trends in other geographic areas. Overall, 33% of victims required substantial subsequent therapeutic clinical resources from our health system beyond a single clinical visit; therefore, the impact of novel e-scooter technology may have been underestimated by early studies of ED visits alone. Our novel methods, using a keyword search and NLP algorithm, allowed identification of e-scooter injuries among more than 36 million electronic medical notes including notes for outpatients who may otherwise have been hidden in electronic health records that cannot easily be queried for diagnostic codes.

By combining multiple health system and public safety data sources, we estimate a utilization-corrected e-scooter injury rate of 115 injuries per million e-scooter trips in our region; this information has heretofore been limited in the existing literature, illustrating the importance of data-sharing. Our estimate of 115 injuries per million trips is of the same order as the limited existing regional studies, but draws from an order of magnitude more injuries and trips than these studies (**Table 2**). Although some existing national database studies have identified similarly large numbers of potentially injured patients, their estimates have varied widely amongst each other, even when using the same data sources, and they have not estimated a utilization-corrected injury rate. In any case, our estimate is substantially higher than prior national estimates of 104 injuries per million motorcycle trips, 15 injuries per million bicycle trips, 8 injuries per million passenger car trips, and 2 injuries per million walking trips [15], although these established statistics are drawn from public safety records that may capture only a more severe subset of injuries. In contrast, our fatality rate of 19 per 100 million e-scooter trips is closer to prior national estimates of 21 per 100 million bicycle trips than 537 per 100 million motorcycle trips [15]. The high prevalence of injuries we find among e-scooter riders may reflect the relative inexperience of riders and difficulty with furnishing and enforcing use of safety equipment among riders using a shared device, along with the rapid uptake of this technology outpacing safety regulations.

**Table 2:** Review of significant prior studies on e-scooter injuries.

Study	Type	Methods	Dates	Population	Sites	Injured Patients Identified	Injury Rate Findings
Ioannides et al	Present study	Chart review (very broad keyword search and NLP algorithm), Assessment of 30-day downstream injury-related medical resource use	January 2014 to May 2020	Outpatient and ED visits in Los Angeles, California, USA	Single large health system, 180 clinics and 2 hospitals	At least 1,354	115 injuries per million trips
Trivedi et al, 2019 [3]	Prior study, peer review, study patients also included in present study	Chart review (simple keyword search)	September 2017 to August 2018	ED visits in Los Angeles, California, USA	Single large health system, 2 hospitals	249	No rate information
Portland Bureau of Transportation, 2019 [16]	Municipal report	Chart review (primarily simple keyword search)	July to November 2018	ED visits and accident reports in Portland, Oregon, USA	All local hospitals	176	251 injuries per million trips
Baltimore City Department of Transportation, 2019 [17]	Municipal report	Chart review (primarily simple keyword search)	August 2018 to January 2019	ED visits and accident reports in Baltimore, Maryland, USA	All local hospitals	63	87 injuries per million trips
San Francisco Municipal Transportation Agency, 2019 [18]	Municipal report	Chart review (primarily simple keyword search)	January 2018 to February 2019	ED visits and accident reports in San Francisco, California, USA	All local hospitals	41	169 injuries per million trips
San Francisco Department of Public Health, 2019 [19]							
Austin Public Health, 2019 [20]	Municipal report; peer review	Chart review (primarily simple keyword search)	September to November 2018	ED visits and accident reports in Austin, Texas, USA	Ambulance records and 9 hospitals	192	200 injuries per million trips
Rix et al, 2021 [21]							
Aizpuru et al, 2019 [22]	Peer review	Review of national database (using diagnosis codes and keyword search)	January 2013 to December 2017	ED visits across the USA	Sample of approximately 100 hospitals	≈32,400 (nationwide weighted estimate)	26 cases per million people
Namiri et al, 2020 [23]	Peer review	Review of national database (using diagnosis codes and keyword search)	January 2014 to December 2019	ED visits across the USA	Sample of approximately 100 hospitals	≈70,644 (nationwide weighted estimate, 988 patients identified)	190 cases per million people, peak rate
Farley et al, 2020 [24]	Peer review	Review of national database (using diagnosis codes and keyword search)	January 2014 to December 2019	ED visits across the USA	Sample of approximately 100 hospitals	≈39,113 (nationwide weighted estimate)	92 cases per million people, peak rate
Traynor et al, 2021 [25]	Peer review	Review of national database (using diagnosis codes and keyword search)	September 2017 to December 2019	ED visits across the USA	Sample of approximately 100 hospitals	≈102,614 (nationwide weighted estimate, 2,754 patients identified)	Increase in share of injury hospitalizations that are related to scooters
Kim et al, 2021 [26]	Peer review	Review of “Emergency Department-based Injury In-Depth Surveillance” database (using diagnosis codes and keyword search)	January 2011 to December 2017	ED visits across South Korea	Sample of 23 hospitals	284	None
Tan et al, 2019 [27]	Peer review	Review of national database (simple keyword search)	January 2015 to December 2017	ED visits across Singapore	All local hospitals	614	“Severe” injury rate 3 times higher in motorized versus non-motorized devices
Blomberg et al, 2019 [28]	Peer review	Chart review (simple keyword search)	January 2016 to July 2019	Emergence dispatches in Copenhagen, Denmark	All local ambulances	468	None
Shichman et al, 2021 [29]	Peer review	Chart review (simple keyword search)	May 2017 to February 2020	ED visits in Tel Aviv, Israel	Single hospital	563 (out of 3,331 potentially injured patients who were excluded)	None
Lavoie-Gagne et al, 2021 [30]	Peer review	Chart review (using scooter diagnosis codes)	November 2017 to March 2020	ED visits in San Diego, California, USA	Single hospital	442	None
Badeau et al, 2019 [31]	Peer review	Chart review (simple keyword search)	January to November 2018	ED visits in Salt Lake City, Utah, USA	2 hospitals	50	None
Dhillon et al, 2020 [32]	Peer review	Chart review (simple keyword search of trauma registry data)	January to December 2018	ED visits in Southern California, USA	9 hospitals	87	None
Vernon et al, 2020 [33]	Peer review	Chart review (simple keyword search)	May 2018 to August 2019	ED visits in Atlanta, Georgia, USA	Single health system	293	None
Moftakhar et al, 2021 [34]	Peer review	Chart review (simple keyword search)	May 2018 to September 2019	ED visits in Vienna, Austria	3 hospitals	175	None
Bekhit et al, 2020 [35]	Peer review	Claims data (simple keyword search)	September 2018 to April 2019	ED visits in Auckland, New Zealand	4 hospitals	770	600 injuries per million trips
Mukhtar et al, 2021 [36]	Peer review	Chart review (simple keyword search)	September 2018 to December 2019	ED visits in Indianapolis, Indiana, USA	Single health system	192	None
Mitchell et al, 2019 [37]	Peer review	Chart review (simple keyword search)	November 2018 to January 2019	ED visits in Brisbane, Australia	Single hospital	54	None
Beck et al, 2020 [38]	Peer review	Chart review (using injury diagnosis codes)	January to February 2019	ED visits in Dunedin, New Zealand	Single hospital	54	None
Cicchino et al, 2021 [39]	Peer review	Prospective registry of ED patients	March to November 2019	ED visits in Washington, DC, USA	Single hospital	99	21 injuries per million miles (3.8 times higher than cyclists)
Heuer et al, 2021 [40]	Peer review	Prospective registry of ED patients	June 2019 to June 2020	ED visits in Hamburg, Germany	Single hospital	90	None
Störmann et al, 2020 [41]	Peer review	Chart review	July 2019 to March 2020	ED visits in Frankfurt, Germany	2 hospitals	76	None
Mair et al, 2021 [42]	Peer review	Chart review	July 2019 to April 2020	ED visits in Munich, Germany	Single hospital	60	None

While this concerning injury rate appears to be decreasing over time, this may be confounded by the COVID-19 pandemic reducing e-scooter use and traffic by other road users. Alternately, e-scooter injury rates may be plateauing or decreasing as road users become more familiar with the presence of e-scooters. We present the first longitudinal data on injury rates, which are consistent with this hypothesis but cannot confirm it.

Our keyword search and NLP-based methods permitted us to examine a vast quantity of more than 36 million clinical notes. Furthermore, these methods enabled us to capture larger public health implications of utilization across the health care spectrum, notwithstanding that our highly specific NLP algorithm had lower accuracy on outpatient notes than ED notes (due to the lower prevalence of e-scooter injury among outpatients). Even if, as we advocate, consistent terminology and diagnostic codes for e-scooter injuries are established, our NLP-based approach may be useful for identifying other novel clinical presentations for which diagnosis codes are unavailable or inconsistently used but where large amounts of unstructured electronic data are available. We chose to combine current state-of-the-art supervised learning techniques in order to maximize accuracy and minimize the amount of training data required (reviewing some very terse medical notes was a key investigational challenge, sometimes requiring specialized training and knowledge of local context and practices). These techniques are well established, and the chief novelty of this study is its clinical application and the consequent results.

Our study has a number of limitations. First, the e-scooter injury rate we report may be conservative as we do not capture injuries treated at other health systems in our area, nor injuries that may never present to care but nonetheless cause temporary or permanent disability, and we did not include 138 probable e-scooter injuries and roughly 500 injuries missed by our NLP algorithm (which would have required manual review of roughly 12,000 clinical notes to confirm). Since our study was retrospective in nature, we did not have standardized data collection, and this limits available clinical information. Reliable assessments of substance use, helmet utilization, and the exact geographic location of injuries would allow for further insights. Finally, some e-scooter injuries may have resulted from individually owned e-scooters rather than devices owned by private operators.

Our study also has a number of unique strengths. Our NLP methods greatly reduced the burden of manual review of clinical notes; for example, 506 injuries were identified from review of 1,029 NLP predicted positive outpatient notes, whereas manual review of around 12,276 outpatient notes would likely have been required using only keyword search to identify the same number of injuries (assuming a 4.1% overall adjusted prevalence of E-scooter injury, compared with 49.2% in the NLP predicted positive data). In addition, whereas previous reports have described only ED visits related to e-scooter injuries, this is the first study to include data on outpatient clinic visits, thus including the full spectrum of care. This is also the first study to provide data on cumulative downstream related healthcare utilization; our finding that about one in three patients received care in multiple health care settings after their injury highlights a larger impact of these injuries than noted in previous reports. We also provide information on trends over time, including during the Covid-19 pandemic, and utilization corrected injury rate per million e-scooter trips.

## Conclusions

E-scooters are associated with injury rates more akin to motorcycles than pedal-driven bicycles. We urge caution about the use of e-scooters and encourage further research into public education, urban planning, and municipal regulation efforts to curb e-scooter injuries. The NLP-based algorithms used to search millions of electronic medical records may increasingly provide a method for rapid identification and assessment of other novel health conditions that do not have established diagnosis codes and tracking systems, which may include infectious diseases as well as traumatic injuries.

## Supporting information

S1 File (DOCX)

## Abbreviations

NLP: Natural language processing
ED: Emergency Department
RMDL: random multi-model deep learning
GloVe: global vectors for word representation
CNN: convolutional neural network
TF-IDF: term frequency-inverse document frequency
DNN: deep neural network
