# Supplementary material for: E-scooter related injuries: Using natural language processing to rapidly search 36 million medical notes
Source: PLoS One. 2022 Apr 6;17(4):e0266097. doi: 10.1371/journal.pone.0266097 (PMC8985928; doi:10.1371/journal.pone.0266097)
Supplement: S1 File — (DOCX) [file pone.0266097.s001.docx]

**SUPPLEMENTARY MATERIALS:**

| **S1 Figure. Flowchart of notes used for our NLP algorithm*** |
| --- |
| **A:**  **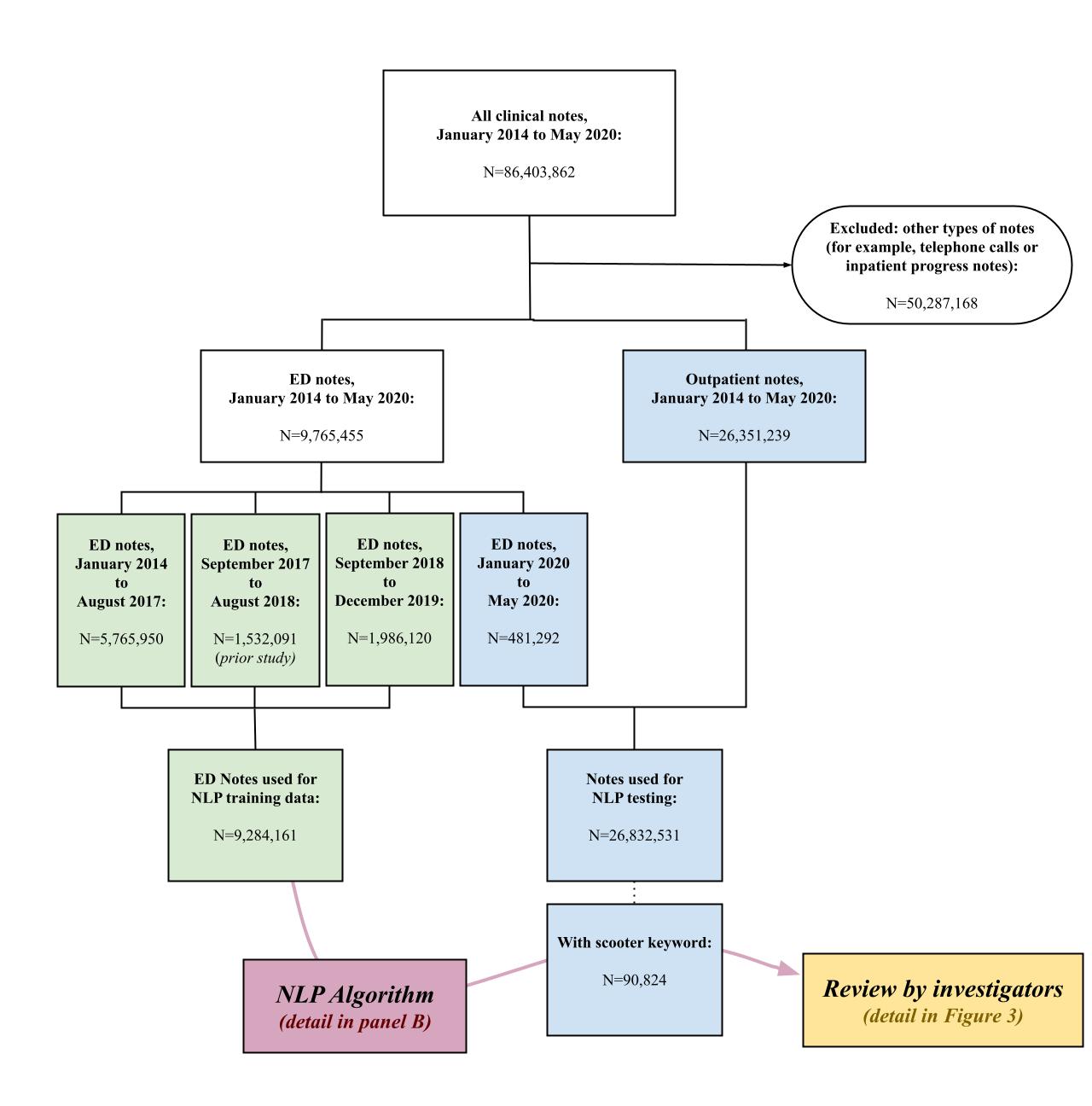** |
| **B:**  **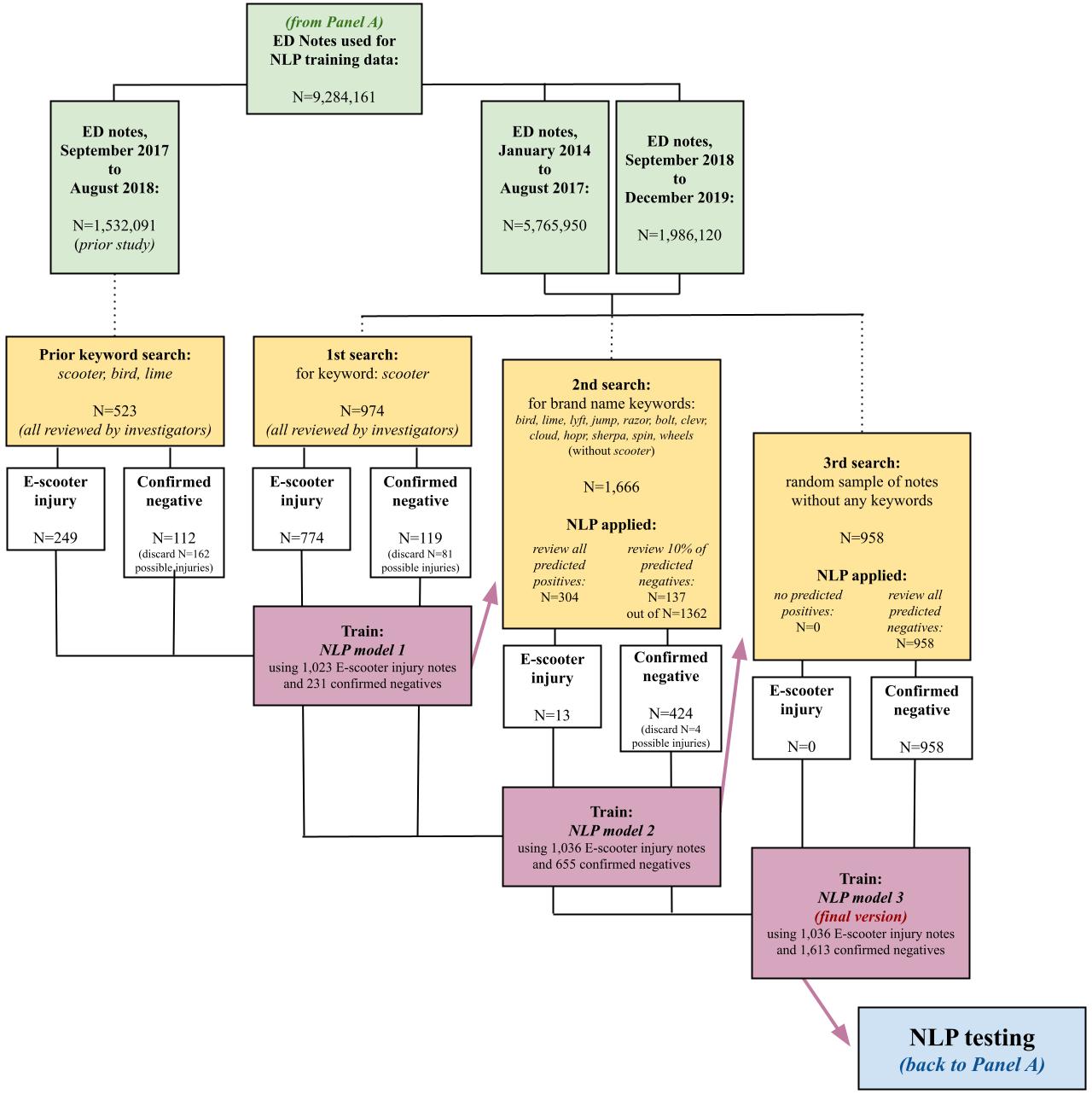** |
| *Panel A details notes used for training data (only ED notes, denoted in green) versus testing data (some ED and some outpatient notes, denoted in blue). NLP algorithm steps are denoted in red, and manual review by investigators in yellow. Panel B (essentially an inset to Panel A) details the iterative process used to assemble our labeled training data using a variety of potential e-scooter keywords in conjunction with preliminary NLP models. All notes in training data were manually reviewed by investigators, and uncertain cases (that neither clearly did nor clearly did not represent an e-scooter injury, denoted as “possible” injuries) were not used for NLP training. “Confirmed Negative” refers to a note that, on review by investigators, does not describe an e-scooter injury; similarly, “Predicted Negative” refers to a note that our NLP algorithm predicted to have a less than 90% probability of describing an e-scooter injury, while “Predicted Positive” refers to a note with a 90% or greater probability. Dashed lines represent steps where some notes were set aside if they lacked keywords. |

| **S1 Table. Definition of e-scooter injury and examples for abstraction** | | | |
| --- | --- | --- | --- |
|  | | | |
| **Question** | **Answers** | **Definition** | **Fictionalized Sample Note** |
| **Was this an e-scooter injury?** | Yes (probable e-scooter related injury; confirmed) | The patient was likely injured as a result of an electric scooter. | "patient was riding a bird electric scooter on wilshire boulevard, turning a corner, and the bird scooter slid so the patient jumped off and landed awkwardly on the left leg" |
|  | No (unlikely) | The patient was not injured as a result of an electric scooter, or not injured at all. | "today she presents for follow-up of her essential hypertension and hyperlipidemia using a knee scooter for assistance” |
|  | Maybe (possible) | It is possible the patient was injured due to an electric scooter, but it is unclear based on available information in clinical notes. | "12 year-old male brought here today my mom for a scooter injury earlier today. hit a pothole, fell off, started crying and began complaining of wrist and knee pain" |
| **Was the patient riding an e-scooter when the incident took place?** | Rider | Patient injury occurred while riding an electric scooter. | "this morning, patient was riding a bird scooter, lost balance and had to suddenly place his left foot down forcibly, twisting the ankle" |
|  | Non-rider | Patient injury occurred due to an electric scooter, but the victim patient was not riding an electric scooter. | "returns for follow-up of a radial head fracture sustained 2 weeks ago when she was hit by a bird scooter, has been wearing a sling and splint and taking tylenol as needed for pain” |

| **S2 Table. Diagnostic performance of NLP algorithm on testing data*** | | | |
| --- | --- | --- | --- |
|  | | | |
|  | **Outpatient notes**  N=2275 | **ED notes**  N=125 |  |
| Injury Prevalence | 8% | 51%  (42-60%) |  |
| Sensitivity | 50% | 67%  (54-78%) |  |
| Specificity | 96% | 93%  (84-98%) |  |
| Positive predictive value | 49%  (47-51%) | 91%  (80-97%) |  |
| Negative predictive value | 96%  (95-97%) | 73%  (66-80%) |  |
| Positive likelihood ratio | 12.0 | 10.3  (3.9-26.8) |  |
| Negative likelihood ratio | 0.5 | 0.4  (0.3-0.5) |  |
| Overall accuracy | 92% | 80%  (72-87%) |  |
| Area under the receiver operating characteristic curve (AUROC) | 0.89 | 0.87  (0.81-0.93) |  |
| *95% confidence intervals are included where calculable; due to the volume of predicted negative notes among outpatients, we only have gold standard assessments by the investigators for a 10% sample of the NLP predicted negatives, thus we only calculate confidence intervals for positive and negative predicted value (see Table S4). Table S4 presents test characteristics where the “possible” e-scooter injuries are re-classified as positives. | | | |

| **S3 Table. Contingency tables for diagnostic performance of final NLP algorithm on ED and Outpatient Notes*** | | | |
| --- | --- | --- | --- |
|  | | | |
| **ED Notes** | **Confirmed  E-scooter Injury** | **No (or “possible”)  E-scooter Injury** |  |
| **NLP Predicted Positive**  (at least 90% probability) | 43  True Positives: classified by both NLP algorithm and human review as an e-scooter injury | 4  False Positives: classified by NLP algorithm as an e-scooter injury but was not an e-scooter injury based on human review |  |
| **NLP Predicted Negative**  (less than 90% probability) | 21  False Negatives: classified by NLP algorithm as not an e-scooter injury but was an e-scooter injury based on human review | 57  True Negatives: classified by both NLP algorithm and human review as not an e-scooter injury |  |
|  |  |  |  |
| **Outpatient Notes** | **Confirmed  E-scooter Injury** | **No (or “possible”)  E-scooter Injury** |  |
| **NLP Predicted Positive**  (at least 90% probability) | 506 | 523 |  |
| **NLP Predicted Negative**  (less than 90% probability) | 50, *scaled=500 (to account for 10% sampling, except for NPV calculations)* | 1,196, *scaled=11,960*  *(to account for 10% sampling, except for NPV calculations)* |  |
|  |  |  |  |
| *Counts for NLP predicted negative outpatient notes were scaled upwards tenfold to account for 10% sampling for manual review to obtain gold standard classifications. 21 “false negative” ED notes and roughly 500 “false negative” outpatient notes were not abstracted and did not contribute to our injury totals. | | |  |

| **S4 Table. Secondary analysis of NLP diagnostic performance under alternate assumption treating possible injuries as e-scooter injuries rather than non-injuries.*** | | | |
| --- | --- | --- | --- |
|  | | | |
|  | **Outpatient notes**  N=2275  *(including 130 possible injuries)* | **ED notes**  N=125  *(including 8 possible injuries)* |  |
| Injury Prevalence | 16% | 58%  (48-66%) |  |
| Sensitivity | 25% | 60%  (48-71%) |  |
| Specificity | 96% | 92%  (82-98%) |  |
| Positive predictive value | 51%  (49-53%) | 91%  (80-97%) |  |
| Negative predictive value | 87%  (86-89%) | 63%  (56-69%) |  |
| Positive likelihood ratio | 5.6 | 7.9  (3.0-20.7) |  |
| Negative likelihood ratio | 0.8 | 0.4  (0.3-0.6) |  |
| Overall accuracy | 84% | 74%  (65-81%) |  |
| Area under the receiver operating characteristic curve (AUROC) | 0.89 | 0.87  (0.81-0.93) |  |
| *As in Table S2, confidence intervals are presented at the 95% level. Diagnostic performance is poorer under this assumption. | | | |

| **S5 Table. E-scooter injuries by calendar year.*** | | | |
| --- | --- | --- | --- |
|  | | | |
| *Year* | Riders (N=1258) | Non-riders (N=96) | **Total (N=1354)** |
| 2014 | 4 | 0 | 4 |
| 2015 | 5 | 0 | 5 |
| 2016 | 13 | 0 | 13 |
| 2017 | 12 | 0 | 12 |
| 2018 | 537 | 58 | 595 |
| 2019 | 641 | 31 | 672 |
| 2020 (through May 14, affected by COVID-19 pandemic) | 46 | 7 | 53 |
| *Yearly totals over complete duration included in study data, including period prior to widespread roll-out of shareable e-scooters in late 2017. | | | |
